# The Deuterator: software for the determination of backbone amide deuterium levels from H/D exchange MS data

**DOI:** 10.1186/1471-2105-8-156

**Published:** 2007-05-16

**Authors:** BD Pascal, MJ Chalmers, SA Busby, CC Mader, MR Southern, NF Tsinoremas, PR Griffin

**Affiliations:** 1 Translational Research Institute, The Scripps Research Institute – Scripps Florida, 5353 Parkside Drive, Jupiter, FL 33458, USA; 2 Department of Biochemistry, The Scripps Research Institute – Scripps Florida, 5353 Parkside Drive, Jupiter, FL 33458, USA; 3 Department of Informatics, The Scripps Research Institute – Scripps Florida, 5353 Parkside Drive, Jupiter, FL 33458, USA

## Abstract

**Background:**

The combination of mass spectrometry and solution phase amide hydrogen/deuterium exchange (H/D exchange) experiments is an effective method for characterizing protein dynamics, and protein-protein or protein-ligand interactions. Despite methodological advancements and improvements in instrumentation and automation, data analysis and display remains a tedious process. The factors that contribute to this bottleneck are the large number of data points produced in a typical experiment, each requiring manual curation and validation, and then calculation of the level of backbone amide exchange. Tools have become available that address some of these issues, but lack sufficient integration, functionality, and accessibility required to address the needs of the H/D exchange community. To date there is no software for the analysis of H/D exchange data that comprehensively addresses these issues.

**Results:**

We have developed an integrated software system for the automated analysis and representation of H/D exchange data that has been titled "The Deuterator". Novel approaches have been implemented that enable high throughput analysis, automated determination of deuterium incorporation, and deconvolution of overlapping peptides. This has been achieved by using methods involving iterative theoretical envelope fitting, and consideration of peak data within expected *m/z *ranges. Existing common file formats have been leveraged to allow compatibility with the output from the myriad of MS instrument platforms and peptide sequence database search engines.

A web-based interface is used to integrate the components of The Deuterator that are able to analyze and present mass spectral data from instruments with varying resolving powers. The results, if necessary, can then be confirmed, adjusted, re-calculated and saved. Additional tools synchronize the curated calculation parameters with replicate time points, increasing throughput. Saved results can then be used to plot deuterium buildup curves and 3D structural overlays. The system has been used successfully in a production environment for over one year and is freely available as a web tool at the project home page .

**Conclusion:**

The automated calculation and presentation of H/D exchange data in a user interface enables scientists to organize and analyze data efficiently. Integration of the different components of The Deuterator coupled with the flexibility of common data file formats allow this system to be accessible to the broadening H/D exchange community.

## Background

Amide hydrogen/deuterium exchange (H/D exchange) coupled with mass spectrometry (MS) has proven to be a powerful technique for the study of protein structure and dynamics as well as protein-protein and protein-ligand interactions [1,2]. The rate at which an amide hydrogen exchanges with solvent deuterium reveals much about its local environment. Changes in protein structure or dynamics can result in changes in H/D exchange kinetics for a subset of amide hydrogens along the protein backbone. Measurement of these alterations in kinetics provides chemical sensors for changes in protein structure and dynamics.

To perform these experiments the target protein or protein complex of interest is subjected to a time course of incubation with deuterium containing buffer to initiate on-exchange of deuterium into the protein. At prescribed time intervals, aliquots of the on-exchange mixture are rapidly quenched by the addition of a low pH and low temperature buffer containing denaturants to prevent further on-exchange, minimize off-exchange of incorporated deuterium and help denature the protein. The quenched and denatured protein is then subjected to enzymatic digestion by acid stable proteases. The resultant peptides are collected on a reverse-phase HPLC column and then gradient eluted directly into a mass spectrometer equipped with an electrospray ionization source where the mass of each peptide is measured. Alternatively, peptides may be spotted onto a target and analyzed with matrix assisted laser desorption ionization (MALDI) mass spectrometry [3,4]. By repeating this process for all on-exchange time points the mass increase of each proteolytic peptide over the time course of deuterium exposure can be determined. From these data the rates of deuterium exchange can be determined for the corresponding areas of the intact protein.

Since the inception of H/D exchange mass spectrometry, substantial advancements in methodology, automation and instrumentation have been made, [5-7] however, processing, curation, and validation of the large number of data points produced in a typical H/D exchange experiment [2,8] presents a significant data processing and analysis challenge. These issues have in part been addressed by several existing software tools and algorithms. Interactive software to simplify the calculation of average molecular weight has been developed [9]. Other algorithms are implemented as command line software which automate the extraction of deuterium content with Fourier transform and natural isotopic abundance based calculations [10,11]. Proprietary software has also been described that automates the extraction of deuterium content in a standalone software application [7,8]. Peak width analysis approaches have been created [12] as well as Excel tools leveraging this approach [13]. Deconvolution techniques have been developed to determine deuterium content in less straightforward situations [14-16]. These above mentioned approaches have shown success in given cases, and their efforts serve to illustrate the interest and challenge of this area, however, the processing and management of H/D exchange data remains a significant bottleneck in the technique.

While progress has been made in H/D exchange data processing, these solutions have not always been easy to employ, and thus have not been widely adopted. Some solutions are command line based, and others are not freely available or their methods have not been described in detail. Often users are required to run separate programs, covering different functions such as peptide location and centroiding. In addition, output from the wide variety of MS instruments and database search engines presents another hurdle in the creation of broadly applicable solutions. There are no published H/D exchange software solutions based on platform independent common mass spectrometry data file formats, such as mzXML. The large size and number of files in conventional studies overburdens users with organizing, migrating and storing data, a critical factor that has yet to be addressed. Integrated user interfaces are also required that allow for the presentation, curation and validation of the results. These factors illustrate the need for software that is precise, efficient, convenient to use, and freely available to the scientific community.

A web-based software system called "The Deuterator" is presented here. It is freely available as an online service, and addresses many of the needs described above. The software can be used to precisely determine the deuterium content for protein segments in an integrated, high-throughput manner, taking steps toward instrument platform independence through the use of common data file formats. Novel tools have been implemented and are accessible through a user interface providing functionality such as the determination of percentage deuterium incorporation for a given peptide. The software is compatible with data from most common database search engines and is compatible with high and low-resolution mass spectral data. Although the software was developed for analyzing LC ESI-MS data, processing data from H/D experiments that do not incorporate a chromatographic step or data that were generated with MALDI MS instruments are supported equally. The output for each peptide included in the experiment is an accurate measurement of deuterium incorporation over time, which can then be plotted as deuterium vs. Log time graphs. Peptide sequences can be positioned next to the protein sequence to display the sequence coverage. Graphical representations of H/D exchange data can then be added by using two-dimensional color maps to represent the data values, which may also be overlaid onto a high-resolution 3D protein structure. To develop and validate this web-based tool, we tested the system in a production environment for over one year with both low (~2000) and high (>60,000) resolution ESI-MS data.

## Implementation

The Deuterator is available as a web-based application. The back-end architecture was built with Java and the online service was implemented with JSP. The MS data viewers were implemented using open source software [17,18]. Mass spectral and search result data are required in native format, and are converted into common file formats. Protein sequence data is required in FASTA format.

The broad range of mass spectrometers and sequence search tools has resulted in a large number of differing file formats, resulting in compatibility problems for many new and existing software packages. This issue has been addressed by recent efforts to create the common file formats mzXML [19] and mzData [20,21] for mass spectrometric data. One such effort [22] has developed the common formats mzXML and pepXML for mass spectral data and peptide identities respectively, and provides freely available mzXML conversion programs for various instrument formats. The Deuterator leverages these files formats and therefore takes steps toward greater compatibility with a broad range of mass spectrometers and protein search engines.

The components of The Deuterator are best viewed in a browser with tabbed browsing functionality, allowing for many time points to be viewed simultaneously. Results from the calculations are saved in a flat file format (CSV), which are easily viewed with applications such as Microsoft Excel.

## Results and discussion

### Workflow

**Figure 1:**
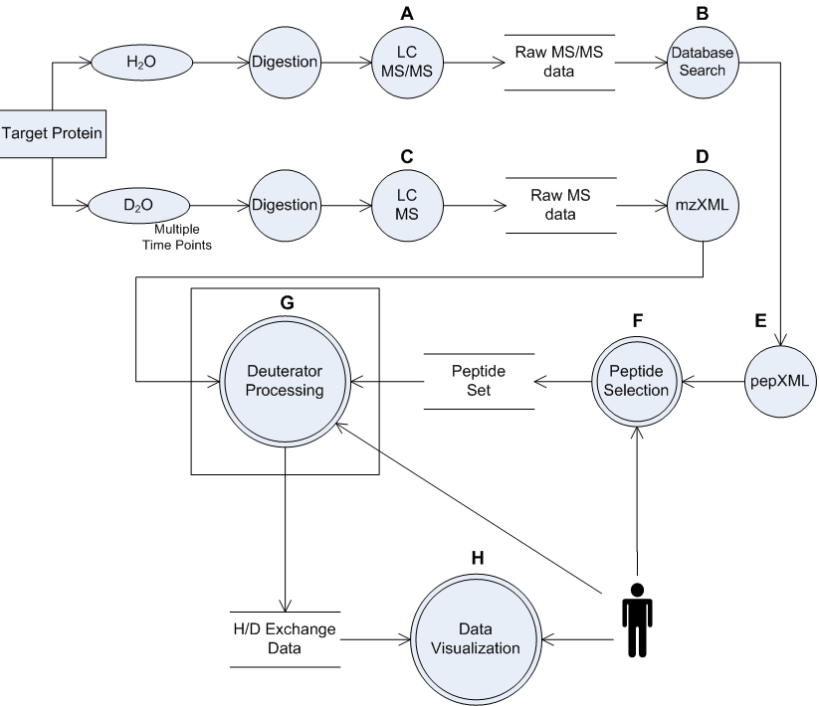
**Data Workflow**. **A**. The protein of interest is digested with an enzyme and the resulting peptides are analyzed by LC MS. **B**. Peptide identity is then established with database search tools such as Sequest. **C**. The protein is incubated with D_2_O at multiple time points, digested with an enzyme and the resulting peptides are analyzed by LC MS. **D**. Resulting raw files are converted to mzXML. **E**. Search results are converted into PepXML. **F**. User selects the peptides of interest from the search results to create the peptide set. **G**. Core Deuterator processing and interactive tools for H/D/exchange data curation. **H**. Data visualization components such as graphs in Microsoft Excel and 3D structure overlays in PyMol.

Our H/D exchange workflow is shown in Figure 1 and starts with a typical proteomics experiment. The protein of interest is digested with an enzyme and the resulting peptides are analyzed by liquid chromatography tandem mass spectrometry (Figure 1A). Peptide identity is then established with the database search tool of choice (*e.g*. X!Tandem, Mascot or Sequest) (Figure 1B) and then visually confirmed with tools such as the Thermo-Finnigan Qual browser. Alternatively, peptides can be identified by using a peptide mass fingerprinting approach. It should be noted that the method for peptide identification, and the stringency by which the results are validated, are left to the end user. Subsequent solution phase amide H/D exchange experiments are performed for a number of pre-defined time periods (typically 8–10 time points spanning 1–10,000 s). Following incubation with heavy water (D_2_O), the protein is digested and the resulting peptides are mass measured with LC-ESI-MS (Figure 1C). The raw MS data and search result files are then converted into the common file formats mzXML and PepXML, respectively (Figure 1D, 1E). The peptides of interest are selected from the search results to create the peptide set (Figure 1F). Some experiments may focus on a narrow region of the protein, in which case sequence coverage may not need to be comprehensive. Other experiments may require full protein sequence coverage, resulting in a larger peptide set. It is for these reasons that the validation and curation of the peptide set is currently the user's responsibility. The Deuterator then retrieves each search result, and uses the protein sequence data and mzXML files to proceed with positional determination, peak identification and centroid calculations (Figure 1G). The details of this method will be further discussed. Resulting data are then made available, and the curated results can then be sent to statistical interrogation and visualization tools (Figure 1H). Third party applications are then used to visualize the results such as graphs in Microsoft Excel and 3D structure overlays in PyMol [23]. Our software facilitates determination of H/D exchange rates and also facilitates differential H/D exchange analysis between multiple treatment conditions (e.g., apo and ligand bound protein).

### Data processing

There are three user inputs into The Deuterator: 1) The mass spectrometry data files (mzXML format); 2) The protein sequence file in FASTA format; and 3) A list of the peptides to be included in the H/D exchange experiment that we refer to as the "peptide set".

**Figure 2:**
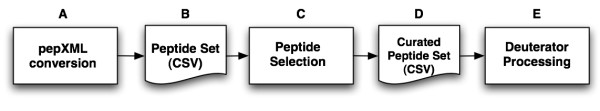
**Peptide Set Process**. **A**. The search results pepXML file is converted to CSV format to facilitate editing. **B**. The raw peptide set in CSV format. **C**. The user selects the peptides of interest. **D**. The final curated peptide set in CSV format. **E**. Deuterator processing.

It is important to note that the comprehensive list of peptide identifications from a database search may not reflect the concise list of peptides of interest for H/D exchange experiments. For instance, some peptide identifications with poor search scores are often not correct, while other peptides identified may lie outside the experimental region of interest. For these reasons, we convert the PepXML search results into the common file format (CSV), and enable manual curation of the "peptide set" prior to import into The Deuterator (Figure 2).

**Figure 3:**
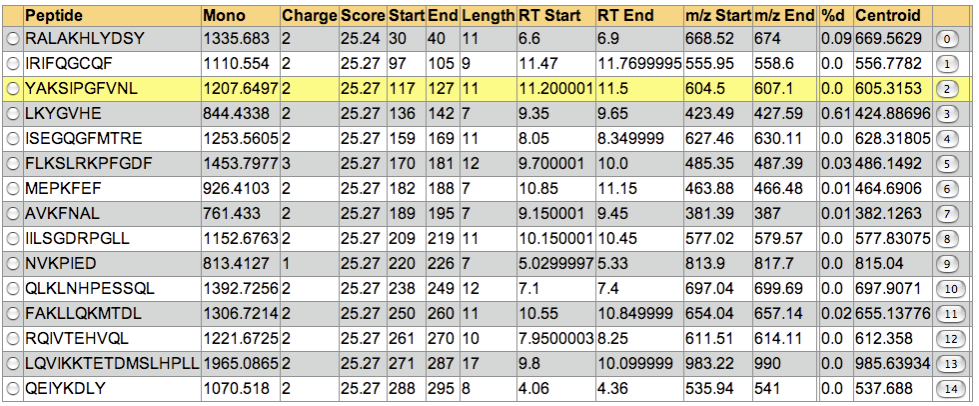
**Data Grid**. Relevant H/D exchange data for each peptide are presented in the grid view. Selection of the peptide loads the associated data into the main spectral and extracted ion viewers.

Once input, the processing begins, and the "peptide set" is initially grouped according to sequence and charge and any redundancy is removed (multiple occurrences of the same peptide ions often occur within a single MS/MS data set). Peptide sequence, charge and retention time are the only data needed for processing, so in cases where these fields are identical, only one row is needed for representation. In cases where there are differing spectrum query times, the average of the group is preserved. Optionally, the score from the peptide search result, if available, can be preserved and represented in the Deuterator interface. The positional information for each peptide is obtained from the protein sequence located within the FASTA file and entered into *start *and *end *data fields. For each peptide the monoisotopic neutral mass is calculated and entered into the Mass data field. This is achieved by the summation of each residue mass, adding the mass of H2O to the result (mass = 18.0106, OH on the C-terminus and the H on the N-terminus). A chromatographic retention time (RT) window for each peptide is defined by *RTStart *and *RTEnd*. These values represent the query-spectrum retention time of the peptide in the pepXML file ± 0.15 minutes and function to allow for some temporal variability in the chromatographic step. All data fields are displayed to the user in the "Grid View" (Figure 3), which also contains additional data fields for each peptide; *MZ*_0_, *MZ*_100_, *MZ*_*Start*_, *MZ*_*End*_, *%D *and *Centroid*.

Accounting for the charge state of the peptide, the 0% deuterium incorporation minimum, *mz*_0_, is defined as being the mass to charge ratio (*m/z*) value for the peptide's monoisotopic m/z. The 100% deuterium incorporation *m/z *limit, *mz*_100_, for the given protein segment is then calculated according to equation where *n *= number of amino acids in the peptide, *p *= the number of non-N-terminal prolines, and *z *= charge.

*mz*_100 _= *mz*_0 _+ ((*n *- *p *- 2)/*z*)

The amino acid proline has no amide hydrogen and so they are subtracted from the equation. The subtraction of 2 in the equation is explained elsewhere [24]. The values of *mz*_0 _and *mz*_100 _are used in subsequent calculations and are presented in the interface to serve as positional markers. These values are different from *MZ*_*Start *_and *MZ*_*End*_, which are the user-defined limits of the *mz *range, used to define the centroid calculation.

**Figure 4:**
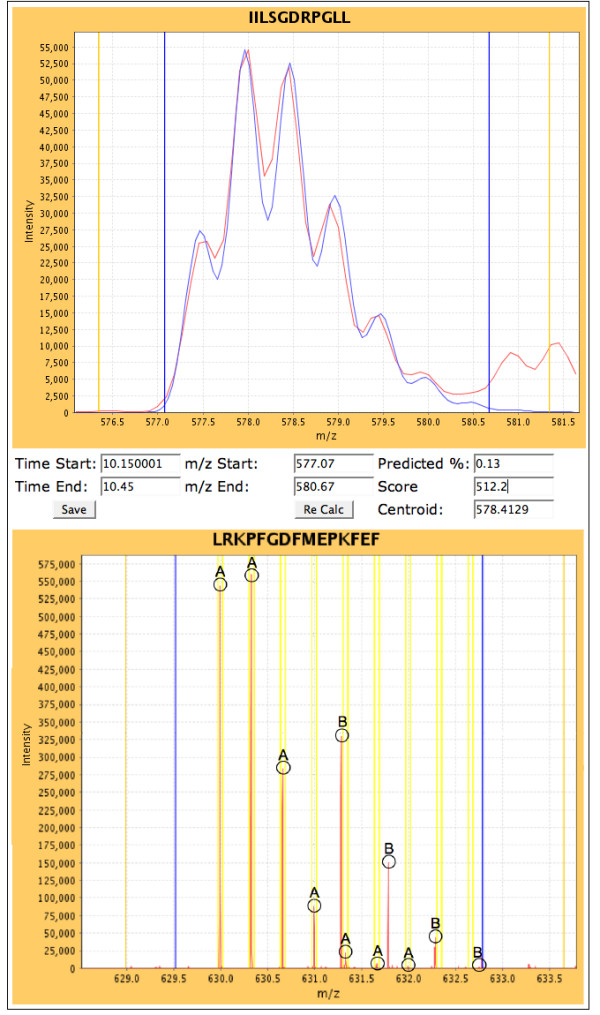
**Main Spectral Viewer**. **Top**. Co-added low-resolution spectral data within the selected retention time range is presented in the viewer. The theoretical isotopic envelope (blue) is overlaid on the observed data (red), showing the best-fit envelope used for calculation of deuterium content. **Bottom**. Co-added high-resolution spectral data within the selected retention time range is presented in the viewer. Correct peak data is extracted from calculated sub-ranges only, displayed as yellow bars. Correct peaks are extracted from the spectral data, enabling more accurate centroid measurements in areas with poor signal to noise ratios or overlapping peptides. 'A' Peaks belong to the peptide of interest and all reside within the sub ranges. 'B' peaks belong to another peptide (+1), which will be disregarded from centroid calculations.

**Figure 5:**
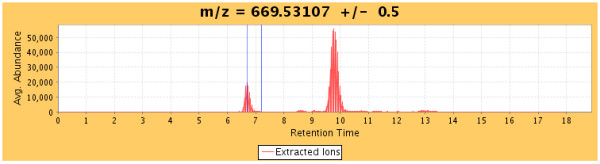
**Extracted ion chromatogram**. The averaged ion intensities for a centroid mass ± 0.5 (Da) are displayed across all scans allowing the user to graphically determine the retention time range that will produce the best data results. In the case above, the co-added data may yield an isotopic profile with a higher signal-to-noise ratio if the retention time start and end positions were reduced slightly.

The software determines the average intensity of observed ions with given *m/z *values within the *m/z *and RT ranges defined by *RTStart *and *RTEnd MZStart *and *MZEnd*. The average intensities of ions with given *m/z *values are derived by dividing the summed total of their observed intensities by the number of contributing spectra in native or profile mode. The end result is a series of mass-intensity pairs, called the "co-added" data that represents the isotopic distribution of the peptide ion of interest. The co-added data is displayed in the appropriate spectral viewer. Figure 4 shows examples of low (top), and high (bottom) resolution data. To assess the accuracy of the RT window we present an extracted ion chromatogram above the spectral viewer (Figure 5).

### Deuterium exchange calculations

The calculation of the amount of backbone deuterium exchange can be accomplished using one of two methods, the "centroid" method or the "theoretical fit method".

**Figure 6:**
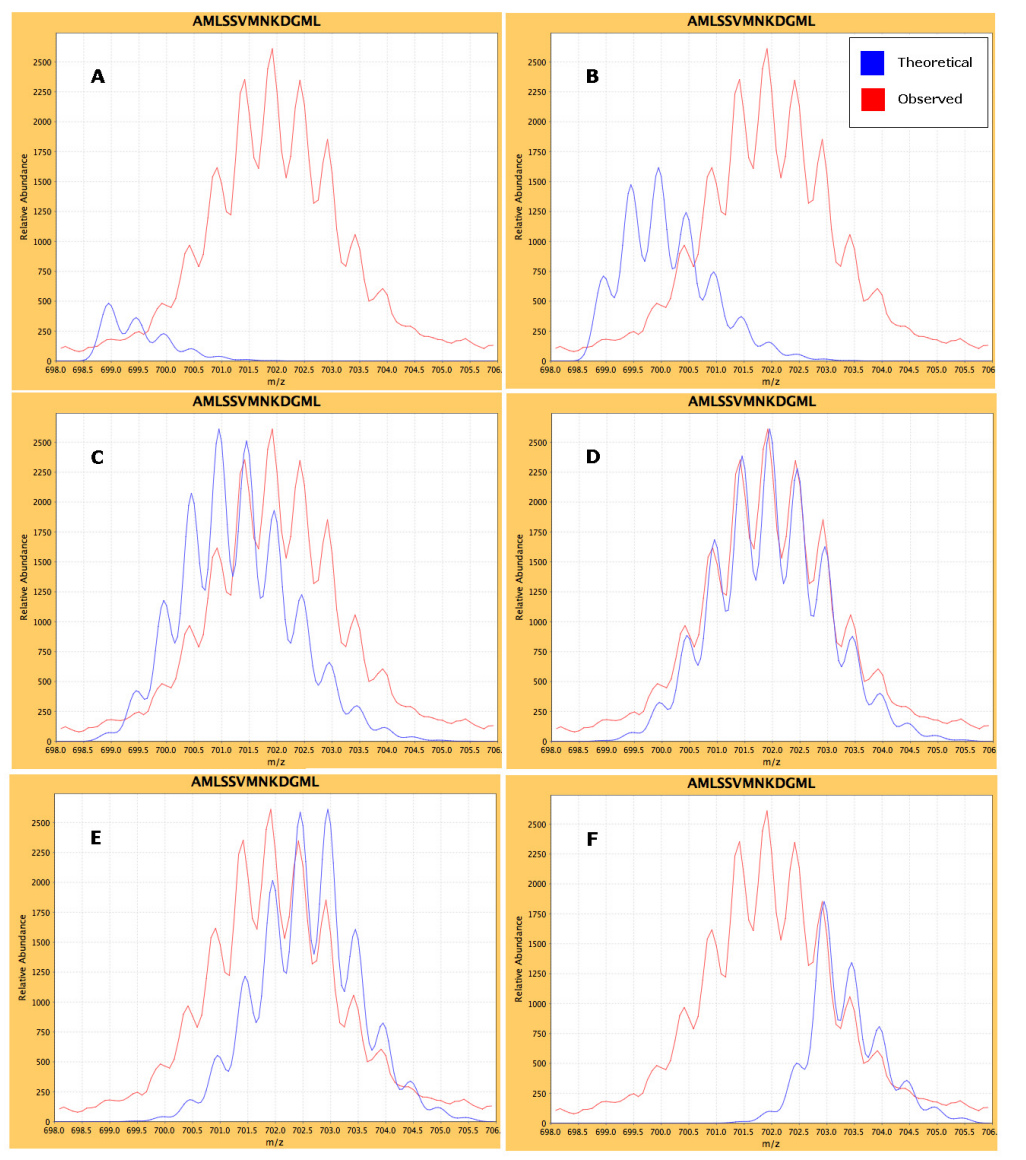
**Iterative Theoretical Isotope Approach**. 1. The natural isotopic envelope is calculated for the peptide at 0% deuterium content and scaled to the highest observed intensity within its m/z range. The chi-square fit score is noted. 2. The process is repeated in 1% increments to 100%. 3. The best (lowest) score is used in the results. The panels above show some of the iterations at increasing levels of deuterium incorporation. A = 0%, B = 10%, C = 25%, D = 43%, E = 65%, F = 90%. The best chi-square score was for panel D at 43% deuterium incorporation.

For the theoretical fit method we followed the approach previously described [11], where the theoretical isotopic envelope is fit to the observed data at varying levels of deuterium incorporation. A number of approaches to calculate the natural isotopic envelope have been described [25-28]. Our method determines the natural isotopic envelope of the undeuterated peptide ion, by first determining its chemical formula, and submitting it to the available software package qmass [26]. The isotopic abundance calculations from this software are based on the natural abundance ratios of each element, which are stored in a file (ISOTOPE.DAT). To produce a distribution representing a fixed percentage of deuterium incorporation, an entry in the isotope distributions file is created to represent the amide hydrogens, which we label as 'D'. The ratios of 'D' are altered to reflect the desired percentage of deuterium incorporation. For example, to find the isotopic distribution for the peptide sequence "FRVSE" at 10% deuterium incorporation, one would first determine the chemical formula for the sequence. The resulting formula (C28H44N8O9) is then modified to account for the number of exchangeable hydrogens (D4), to C28*H40D4*N8O9. The ^1^H to ^2^H ratio is then changed to 0.9/0.1, reflecting the 10% deuterium incorporation. The resulting qmass theoretical distribution is then scaled to the observed high peak intensity within the predicted theoretical *m/z *range, smoothed using the normal distribution formula, and aligned according to the resolution of the instrument. Least squares is then used to fit the envelope to the observed spectral data by measuring the difference between the Y coordinates (intensity) of the theoretical and observed plots, squaring them, and summing the result of all Y pairs. This process is repeated, iterating through increasing levels of deuterium incorporation (Figure 6), and the lowest score is assumed to be the best fit. The percent deuterium incorporation that produced the best fit is populated into the *%D *field within the data grid and can be used for downstream calculations. This approach has been incorporated for low-resolution data analysis and is currently being implemented for high-resolution data.

A different approach is taken for the "centroid" method. Variables *m/z minimum *and *m/z maximum *are initially assigned to *mz*_0 _-1 Da and *mz*_100 _+1 Da, and are adjustable. XY data between *m/z minimum *and *m/z maximum *are used to calculate the intensity weighted mean *m/z centroid *value. The final centroid result is then used to calculate the percentage of deuterium incorporation with methods outlined previously [29].

For high resolution MS data, the isotopic peaks can be expected to reside within much narrower *m/z *ranges allowing for precise determination of the weighted average m/z value. Peptide ion isotopes are spaced at equal *m/z *steps according to the charge of the ion. For a doubly charged ion, its isotopes are spaced 0.5 m/z apart and for a triply charged ion, its isotopes are spaced 0.33 m/z apart. Combining this information with the known monoisotopic mass and charge state of the peptide ion allows the algorithm to predict the central m/z of the first isotope in the distribution, as well as the m/z spacing of the isotopic cluster. An approach using *m/z *ranges, which we will henceforth call "*m/z *sub-ranges", is presented here, where the algorithm only considers data residing within a predefined *m/z *distance from the expected peptide ion center. The sub-range centers are defined by the following equation:

fi=f0+izi=0,...,imax
 MathType@MTEF@5@5@+=feaafiart1ev1aaatCvAUfKttLearuWrP9MDH5MBPbIqV92AaeXatLxBI9gBaebbnrfifHhDYfgasaacH8akY=wiFfYdH8Gipec8Eeeu0xXdbba9frFj0=OqFfea0dXdd9vqai=hGuQ8kuc9pgc9s8qqaq=dirpe0xb9q8qiLsFr0=vr0=vr0dc8meaabaqaciaacaGaaeqabaqabeGadaaakeaadaWfqaqaaiabdAgaMnaaBaaaleaacqWGPbqAaeqaaOGaeyypa0JaemOzay2aaSbaaSqaaiabicdaWaqabaGccqGHRaWkdaWcaaqaaiabdMgaPbqaaiabdQha6baaaSqaaiabdMgaPjabg2da9iabicdaWiabcYcaSiabc6caUiabc6caUiabc6caUiabcYcaSiabdMgaPnaaBaaameaaieGacqWFTbqBcqWFHbqycqWF4baEaeqaaaWcbeaaaaa@4488@

where *f*_0 _= *mz*_0_, *z *= charge state, *i *is an integer, and *i*_*max *_is defined as:

*i*_*max *_= *z *(*α *- *f*_0_)

where *α *= end *m/z*. The resulting windows are set to a width based on the desired distance from the peptide ion center to accommodate for the resolution of the instrument. A 0.025 *m/z *distance has been shown to be effective for a resolution of ~60,000, that generates a baseline peak width of ~0.015 *m/z*. The Deuterator uses this number as default. Ions with *m/z *values outside of these sub-ranges cannot arise from the peptide ion of interest and thus they are removed from further consideration. The weighted average of the data within these expected *m/z *sub-ranges is then calculated. The interface also allows the limits of the *m/z *sub-ranges to be adjusted, which enables the correct peaks to be extracted from the spectral data. This approach has been shown to correctly distinguish peptides in complex situations such as overlapping peptides or peptide ions with poor signal-to-noise (S/N) ratios. The limits of *m/z *sub-ranges are displayed in the user interface as yellow bars, allowing the visual confirmation of the approach (Figure 4 bottom).

### Main analysis page

It is often beneficial to visually validate and manually curate the data. The main analysis page provides the necessary tools to facilitate the validation, curation and recalculation of the *%D *and *Centroid *values. This page has three sections: the data grid, the spectral viewer, and the extracted ion chromatogram.

When a peptide row is selected from the data grid, the co-added spectral data for the calculated *m/z *range of the peptide are presented in the main spectral viewer (Figure 4). The best-fit theoretical isotopic envelope is then overlaid and the sub-ranges are presented for high-resolution data. The indicator bars display the *mz*_0_, *mz*_100_, *m/z*, and centroid range positions. Adjustment of the *m/z *range controls allow for zooming in and out of the spectral data. Navigation controls are available to further refine the sub-range widths (for high resolution data), retention time range, and centroid range of the selected peptide, with the *%D *and *centroid *values being automatically recalculated after each refinement. The extracted ion chromatogram (Figure 5) is also populated when a peptide is selected, and the averaged ion intensities for a given mass across all scans is presented. Blue bars display the retention time ranges used for the extracted ion plots, and can be adjusted for refinement of the scan range with the best abundance for a given peptide's centroid mass. For example, when the retention time start and end values are equal, the view will represent spectral data from the nearest single scan. When the user has completed data curation, the results can be saved.

### Synchronization function

Adjusting data in the main page can be a lengthy task, especially if there are a large number of curations needed for several experimental repetitions. Retention time and *m/z *ranges for like peptides across replicates are usually consistent. By synchronizing across the replicate data sets the number of manual curations needed is significantly reduced and automated reprocessing of centroid calculations is enabled. The synchronization page provides this functionality by allowing the selection of a "core' time point, a list of "sync" time points, and data column values. The process then adjusts the appropriate ranges and the centroids are recalculated for each peptide row. This has demonstrated to save substantial amounts of time by automating the curation process. A typical experiment produced approximately 100 peptides at eight timepoints. Each experiment was replicated four times for consistency. In this case 3200 peptides, each taking one minute to curate with the software, would cost the end user around 53 hours. In our tests, the synchronization function has been shown to be effective on roughly 20% of peptides saving over 10 hours of manual curation time.

### Output

**Figure 7:**
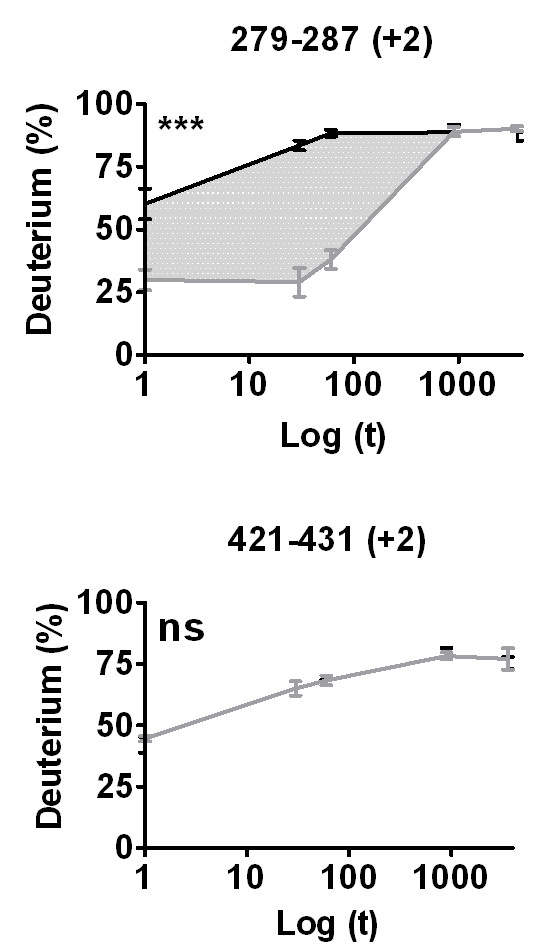
**Deuterium vs. Log Time (s)**. Differential H/D exchange data can be exported and plotted as %D vs. Log time (s) plots. Here we show data from a differential H/D exchange experiment to investigate perturbation in H/D exchange rates upon binding of a ligand (rosiglitazone) to PPARγ. Black line = PPARγ, Grey line = PPARγ + ligand. **Top**. The region between residues 279 and 287 exhibits a significant slow down in exchange rate following ligand binding. **Bottom**. Exchange rates for the region spanning residues 421–431 show no change. Statistical summary from a 2-way ANOVA between apo and ligand bound data; *** = P < 0.001, ns = not significant.

**Figure 8:**
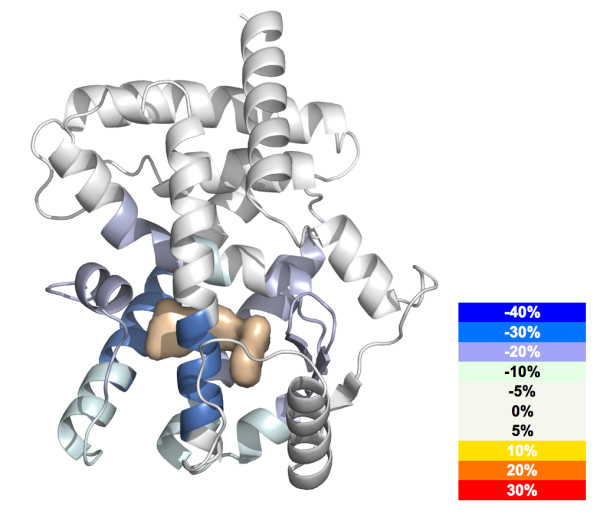
**3D Overlay**. H/D exchange data can be plotted over the three-dimensional protein structure. Here we show the % difference in H/D exchange rate for PPARγ following binding of rosiglitazone plotted over 2PRG.pdb (ligand is represented as a surface rendering). The magnitude of any change in H/D exchange rate upon ligand binding is plotted according to the color key.

Once the *Centroid *and *%D *values for all of the peptides in the peptide set have been determined and saved, the entire grid data can be exported to a common file format. From this point the deuterium content of each peptide is readily calculated using known algorithms [29]. Generation of deuterium buildup curves using tools such as Microsoft Excel (Figure 7) is facilitated. High-resolution 3D structural overlays (Figure 8) can be accomplished using tools such as PyMol [23].

### The Deuterator in practice

The Deuterator has been used extensively on a number of projects over the last year, one of which has been published [30]. Another example of these projects was the measurement of perturbation of H/D exchange rates for the nuclear receptor PPARγ upon binding of synthetic and endogenous ligands. The aim of this research was to establish the different mechanisms of activation of the receptor upon binding of full and partial agonists. The system has been tested with high and low-resolution raw mass spectral data from Thermo Electron LTQ Orbitrap (resolution ~60,000) as well as LTQ linear Ion-Trap (resolution ~1,500). The centroid calculation is a simple weighted average function, and so the validation of the centroid results has been straightforward. The software has been compared to existing tools such as Magtran [9] and the results have been shown to be identical. Native search engine data from the database search programs Mascot and Sequest, and X!Tandem have also been used with the system. The in-house installation is tightly integrated with commercial LIMS software (Proteus, Genologics, Canada), which captures the project files and streamlines automated processing.

### Future work

Rendering components will be built that provide extended graphical capabilities, 2D and 3D color gradient maps, and data grids that convey changes in deuterium incorporation between different experiments. Sequence coverage can be presented in the user interface with heat maps, color-coding the rate of exchange. Improvements in the preprocessing phase will be implemented which will improve precision and reduce further the need for manual intervention. Ongoing tests to increase compatibility with other instrument data formats will be conducted. Any future developments to The Deuterator will be made accessible to the community.

## Conclusion

The Deuterator is a web application presenting integrated and novel functionality in H/D exchange analysis and it is compatible with multiple file formats. The interface presents the centroid results and associated data for each peptide and allows for "drilling-down" and visualization of the mass spectral data. Developed to aid scientists at The Scripps Research Institute in the analysis of H/D exchange data, it is freely available online and eventually will be offered as a standalone open source solution.

## Availability and requirements

Project name: The Deuterator

Project home page: 

Operating system: Platform Independent

Programming language: Java/JSP

Other requirements: N/A

License: available free of charge to academic, government and non-profit users for non-commercial use

## Abbreviations

Da – Dalton

ESI – Electrospray ionization

H/D exchange – Hydrogen/deuterium exchange

JSP – Java Server Pages

LC – Liquid chromatography

MS – Mass spectrometry

*m/z *– Mass-to-charge ratio

NMR – Nuclear magnetic resonance

RT – Retention time

## Competing interests

The author(s) declare that they have no competing interests.

## Authors' contributions

BDP developed the software and wrote the manuscript. MJC provided core requirements and feedback. SAB tested the software and provided requirements. MJC and SAB acquired all the H/D exchange test data sets. CCM managed the overall software development process and design for the project, and assisted with development. MRS contributed development to the implementation of the external website as well as Excel files for rendering H/D exchange data. NFT provided essential direction and oversaw all informatics aspects of the project. PRG conceived and initiated the H/D exchange program, and was the project manager driving the development of the application. All authors have read and approved of the manuscript.

## References

[B1] Engen JR, Smith DL (2001). Investigating protein structure and dynamics by hydrogen exchange MS.. Analytical Chemistry.

[B2] Englander SW (2006). Hydrogen Exchange Mass Spectrometry: A Historical Perspective. Journal of the American Society for Mass Spectrometry.

[B3] Buijs J, Costa Vera C, Ayala E, Steensma E, Hakansson P, Oscarsson S (1999). Conformational stability of adsorbed insulin studied with mass spectrometry and hydrogen exchange.. Analytical Chemistry.

[B4] Mandell JG, Falick AM, Komives EA (1998). Measurement of Amide Hydrogen Exchange by MALDI-TOF Mass Spectrometry. Analytical Chemistry.

[B5] Chalmers MJ, Busby SA, Pascal BD, He Y, Hendrickson CL, Marshall AG, Griffin PR (2006). Probing protein ligand interactions by automated hydrogen/deuterium exchange mass spectrometry.. Analytical Chemistry.

[B6] Dai SYF (2006). Accuracy of SUPREX (Stability of Unpurified Proteins from Rates of H/D Exchange) and MALDI Mass Spectrometry-Derived Protein Unfolding Free Energies Determined Under Non-EX2 Exchange Conditions. J Am Soc Mass Spectom.

[B7] Woods VLJ (2001). High resolution, high-throughput amide deuterium exchange-mass spectrometry (DXMS) determination of protein binding site structure and dynamics: utility in pharmaceutical design. Journal of Cellular Biochemistry.

[B8] Hamuro Y, Coales SJ, Southern MR, Nemeth-Cawley JF, Stranz DD, Griffin PR (2003). Rapid analysis of protein structure and dynamics by hydrogen/deuterium exchange mass spectrometry. Journal of Biomolecular Techniques: JBT.

[B9] Zhang ZM (1998). A Universal Algorithm for Fast Automated Charge State Deconvolution of Electrospray Mass-to-Charge Ratio Spectra. Journal of the American Society for Mass Spectrometry.

[B10] Hotchko M, Anand GS, Komives EA, Ten Eyck LF (2006). Automated extraction of backbone deuteration levels from amide H/2H mass spectrometry experiments. Protein Science.

[B11] Palmblad M (2001). Automatic Analysis of Hydrogen/Deuterium Exchange Mass Spectra of Peptides and Proteins Using Calculations of Isotopic Distributions. Journal of the American Society for Mass Spectrometry.

[B12] Weis DD, Hotchko M, Wales TE, Ten Eyck LF, Engen JR (2006). Identification and characterization of EX1 kinetics in H/D exchange mass spectrometry by peak width analysis.. Journal of the American Society for Mass Spectrometry.

[B13] Weis DD, Engen JR, Kass IJ (2006). Semi-Automated Data Processing of Hydrogen Exchange Mass Spectra Using HX-Express.. Journal of the American Society for Mass Spectrometry.

[B14] Abzalimov RR, Kaltashov IA (2006). Extraction of Local Hydrogen Exchange Data from HDX CAD MS Measurements by Deconvolution of Isotopic Distributions of Fragment Ions.. Journal of the American Society for Mass Spectrometry.

[B15] Chik JK, Vande Graaf JL, Schriemer DC (2006). Quantitating the statistical distribution of deuterium incorporation to extend the utility of H/D exchange MS data.. Analytical Chemistry.

[B16] Zhang Z, Li W, Logan M, Li M, Marshall AG (1997). Human recombinant [C22A] FK506-binding protein amide hydrogen exchange rates from mass spectrometry match and extend those from NMR. Protein Science.

[B17] JFreeChart.

[B18] CEWolf.

[B19] Pedrioli PG (2004). A common open representation of mass spectrometry data and its application to proteomics research. Nature Biotechnology.

[B20] Orchard S, Hermjakob H, Binz PA, Hoogland C, Taylor CF, Zhu W, Julian RK, Apweiler R (2005). Further steps towards data standardisation: the Proteomic Standards Initiative HUPO 3(rd) annual congress, Beijing 25-27(th) October, 2004. Proteomics.

[B21] Orchard S, Hermjakob H, Julian RK, Runte K, Sherman D, Wojcik J, Zhu W, Apweiler R (2004). Common interchange standards for proteomics data: Public availability of tools and schema. Proteomics.

[B22] The Sashimi Project.

[B23] Pymol.

[B24] Bai Y, Milne JS, Englander SW (1993). Primary Structure Effects on Peptide Group Hydrogen Exchange. Proteins: Struc Funct, Genet.

[B25] Clauser KR, Baker P, Burlingame AL (1999). Role of accurate mass measurement (+/- 10 ppm) in protein identification strategies employing MS or MS/MS and database searching.. Analytical Chemistry.

[B26] Rockwood AL, Haimi P (2006). Efficient calculation of accurate masses of isotopic peaks. Journal of the American Society for Mass Spectrometry.

[B27] Rockwood AL, Van Orman JR, Dearden DV (2004). Isotopic compositions and accurate masses of single isotopic peaks.. Journal of the American Society for Mass Spectrometry.

[B28] Yergey JA (1983). A general-approach to calculating isotopic distributions for mass spectrometry. Int J Mass Spectrom Ion Proc.

[B29] Zhang ZS (1993). Determination of amide hydrogen exchange by mass spectrometry: a new tool for protein structure elucidation. Protein Science.

[B30] Quint P, Ayala I, Busby SA, Chalmers MJ, Griffin PR, Rocca J, Nick HS, Silverman DN (2006). Structural mobility in Human manganese superoxide dismutase. Biochemistry.

